# Comparison of Digital Radiography, Conventional Film and Self-Developing Film for Working Length Determination

**DOI:** 10.22037/iej.v13i3.19355

**Published:** 2018

**Authors:** Manucher Raees Sameye, Amin Mohammad Bahalkeh, Arash Izadi, Ania Jafaryan

**Affiliations:** a * Dental Research Center, Golestan University of Medical Sciences, Gorgan, Iran; *; b * Dental School of Islamic Azad University, Tehran, Iran *

**Keywords:** Conventional Radiography, Digital Radiography, Self-developing Film, Working Length

## Abstract

**Introduction:**

Accurate measurement of working length of the root canal is an important factor in endodontic treatment, because it determines the level of cleaning and shaping of the canal. This can be performed using numerous methods including conventional, digital and self-developing methods, which are studied in this work.

**Methods and Materials:**

In this study, 50 maxillary molars with appropriate conditions for the analysis were collected and their mesiobuccal canal lengths were estimated by three different types of radiographs with and without file. Next, two endodontists and a radiologist reviewed all the images under the same conditions. The precise lengths of the canals were measured by removing teeth from their casts and direct observation. Finally, data regarding differences in radiographic length and actual length were examined by SPSS 16.0 software and Repeated Measures ANOVA test.

**Results:**

There was no significant difference in any of the radiographic states. The differences of root canal lengths were not significant for the first (endodontist) and third (endodontist) observers; whereas, there were significant differences for the second observer (radiologist). The differences were not significant for samples without files (*P*=0.89). However, the differences were significant for samples with files (*P*=0.03).

**Conclusion:**

Since analysis showed that there were no significant differences between the results of digital radiography, conventional film and self-developing film methods in working length determination, the clinician can choose any of these methods according to the working conditions without being concerned about losing the accuracy.

## Introduction

Cleaning and shaping of root canal system is a vital aspect of the root canal treatment. Therefore, estimation of the root canal length is necessary before its preparation. Failure in measuring working length could lead to bad consequences such as pain and discomfort of the patient, need for retreatment, root end resection and even tooth extraction [1]. The desired working length for the biometrical preparation and resultant obturation of root canal system is one of the most important phases of endodontic treatment [2].

Various methods are used to determine endodontic working length, such as manual tactile sensation, electronic apex locator, radiography and patient’ reaction. Manual tactile sensation, is the oldest technique in working length determination, which requires a learning curve to achieve expertise [3].

Determination of working length by apex locator has also, been reported to have met with great success, and it also omits the need of radiation and thereby its hazards. But high cost of apex locator and its operational technique act as barriers for its general usage [1, 4]. 

Conventional intraoral imaging is another commonly used modality for working length determination; however, it has its own shortcomings such as two-dimensional replication of a three-dimensional object, possibilities of size and shape distortion, need for a darkroom and complex equipment and need for high level of x-ray exposure [5]. However, introduction of digital intraoral imaging reported to be superior by providing the operator convenience and reducing patient radiation exposure [6]. Usage of direct digital radiography has increased during the recent years. In this method, the radiation level to the patient is reduced from 50% using D or E films, which results in lower hazards of this method [7]. Quality of the digital images could be enhanced by increasing contrast and density of the monitor [8]. Also, there is no need for performing the fixing and developing stages in this method [9]. According to the study conducted by Martínez-Lozano proved accuracy of conventional and digital imaging was 50.6% and 61.4%, respectively, in establishing the true working length [10].

In recent years an intra-oral film, called self-developing film, has become popular. It has advantages such as short developing time and ease of accessibility [11]. At one end of the pocket of self-developing films, a fixing and developing bag exists. After radiation, squeezing this bag drives solution toward the radiographic film which fixes and develops the film. In recent years utilizing achievements of radiography and its different method has turned to a significant preference in dental clinics [3].

The aim of the present study is to make comparison of digital radiography, conventional radiography, and self-developing film for working length determination.

## Materials and Methods

In this study, fifty extracted maxillary molars were selected, all teeth had completely closed apices, no blockage or calcification in the root canals, no root fracture or root decay, no internal or external resorption, no extreme curvature in the roots (less than 45 degrees).

This study was done on mesiobuccal root of maxillary first molars. Selected teeth were stored in 5% sodium hypochlorite for 30 min. All teeth were placed in an autoclave were then washed with 0.9% normal saline.

The teeth were randomly encoded and assembled. They were mounted into the cast. At the beginning, estimated working length was determined based on overlapping file on the film for conventional and self-developing films. Regarding the digital films, the estimated length was determined using a simple image processing. By this scheme, we made sure that all of the files reached to the 0.5 mm before the end of radiographic apex. Next, an access cavity was created by diamond bur. Finally, a #15 K-File file (Dentsply Maillefer, Ballaigues, Switzerland) was places into the canal and the file handle was fixated at its place.

Next, to estimate the length of the canal for each tooth, three radiography methods were used: conventional radiography (E speed, Carestream, USA), self-developing film (Ergonom-X, Dentalfilm, Torinese,Italy), and digital radiography (Suni Medical Imaging Inc, San Jose, CA, USA). All radiographic methods were performed in parallel technique. 

A radiographer took the image with a conventional film and a radiation time of 0.3 sec, 70 kVp, 8 mA and a short-cone 8-inch tube. It was developed in an automatic processor. In the same geometric conditions, radiographer with the self-developing film provided the second radiography. After radiation, the film was developed by pressuring and directing the solution around the film and moving it with the fingers for 2 min. 

Direct digital radiography was also performed with a radiation time of 0.08 sec and the same conditions as the previous radiographies. The images were developed by the software associated with the system DrSuni software (Suni Medical Imaging Inc, San Jose, CA, USA). 

**Table 1:** Comparison of the average difference in three methods of radiography with and without file

**Usage of file**	**Method of radiography (N) **	**Mean(SD) of Length**	**Difference between methods and the golden standard level**
**With File**	**Conventional (50)**	20.35(1.75)	0.64±0.63
	**Digital (50)**	20.58(1.69)	0.79±0.78
	**Self-developing (50)**	20.47(1.83)	0.59±0.59
**Without file**	**Conventional (50)**	20.35(1.74)	0.89±0.93
	**Digital (50)**	20.59(1.69)	0.86±0.95
	**Self-developing (50)**	20.49(1.84)	0.83±0.90
**Total**	**Conventional (100)**	20.47(1.75)	0.64±0.63
	**Digital (100)**	20.48(1.75)	0.79±0.78
	**Self-developing (100)**	20.47(1.75)	0.59±0.59

**Table 2:** Comparison of the mean of the difference between the three radiography method and the golden standard level measured by different observers

**Method of radiography**	**Observer**	**Mean (SD) of the difference with the golden standard**	**Difference between methods and the golden standard level**
**Conventional**	**First observer (endodontist) **	0.65 (0.74)	0.74 (0.65)
**Digital**		0.68 (0.82)	0.82 (0.68)
**Self-developing**		0.64 (0.71)	0.71 (0.64)
**Conventional**	**Second observer (radiologist) **	0.80 (0.67)	0.67 (0.80)
**Digital**		1.13 (0.85)	0.85 (1.13)
**Self-developing**		0.80 (0.72)	0.72 (0.80)
**Conventional**	**Third observer**	0.89 (0.92)	0.92 (0.89)
**Digital**		0.78 (0.76)	0.76 (0.78)
**Self-developing**		0.80 (0.87)	0.87 (0.80)

**Figure 1:**
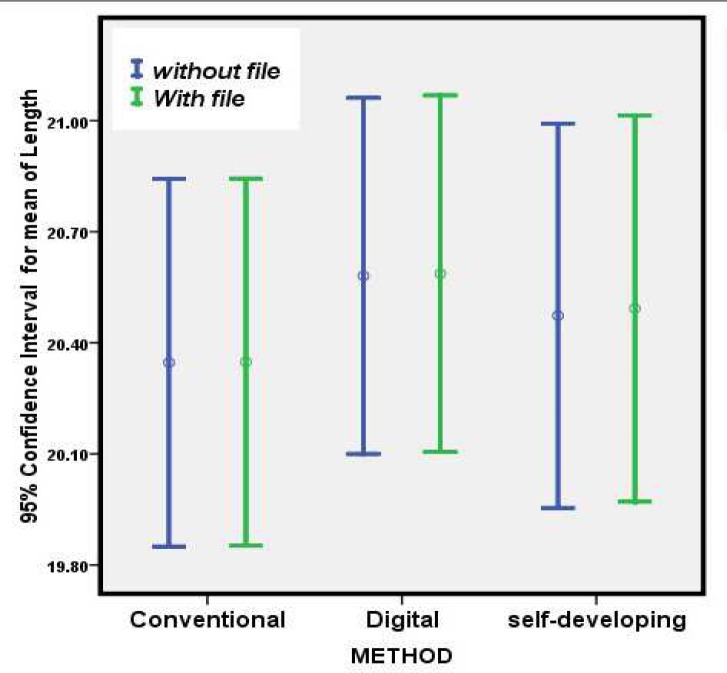
Confidence intervals of the observed root canal lengths

Next all the images were reviewed by two endodontists and a radiologist under the same conditions on the Negatoscope and on the computer monitor. Finally, after observing the film we increased, decreased or kept unchanged the working length. Afterward, samples were removed from the casts. The recorded standard length was determined by reducing 0.5 mm of tip of the file (observed under microscope with ×30 magnification)

Finally, the difference between the estimated lengths in each method was calculated by the golden standard. These differences were evaluated by the repeated measures ANOVA test of the SPSS software (SPSS, version 16.0, SPSS, Chicago, IL, USA). The significant levels were less than 0.05.

## Results

In this study, selected teeth were mounted in their casts and three images were taken from each tooth by the conventional, self-developing and digital radiography. Additionally, for each tooth access cavity was prepared, a #15 K-File was put inside the canal and three radiographies were taken. Finally, root canals lengths were measured using golden standard method explained earlier. The following result were obtained.

According to Table 1, there was no significant difference in any of the radiographic states with and without file. The confidence intervals of the observed root canal lengths are shown in Figure 1. It says that all three radiographic techniques have the same precision.

In Table 2, the root canal length differences between three radiographic methods and golden standard level measured by three observers are shown. The differences of root canal lengths were not significant for the first (endodontist) (with *P*=0.9) and third (endodontist) (with *P*=0.6) observers; whereas, there were significant for the second observer (radiologist) (with *P*=0.02).

## Discussion

Precise working length measurement is an important factor in evaluating the success of an endodontic treatment. Inaccurate measurements cause overfilling, perforation, and higher possibility of pain after the treatment [12, 13]. 

In this study, 50 first maxillary molars were investigated and statistical analysis showed that in general there were no significant differences in measuring the root canal length, between the measurements of these three radiographic methods and the actual length. 

Image magnification due to non-uniform spaces respect to the center of a radiographic tube and image distortion coming from distances between films and the objects are the main affecting parameters to the accuracy of the radiographic images [11]. In the present study, all images were prepared so that the center of object, tube and film placed on a straight line and distance between object and the film become minimal. 

Leddy *et al. *[14] interpreted the endodontic file lengths using RadioVisioGraphy. Their results showed no significant difference in the ability of endodontists to make accurate file length adjustments using conventional radiography versus radiovisiography [14].

Shearer *et al. *[15] stated that there was no statistically significant difference between the percentage of length of root canal visible on conventional film and that visible on RadioVisioGraphy images. Thus, radiovisiography may be considered to be of equal value to conventional film radiography for the imaging of root canal systems *in vitro *[15].

Eikenbergetet *et al. *[16] showed that there were no significant differences between different techniques in detecting apical file position and the technique that is quicker, has less cost, and no need for manual fixing and developing is always the most desired approach. Our study is consistent with their study in finding no significant differences between conventional, digital and self-developing methods.

A previous study showed that changing the diameter of the files deteriorates accuracy of the measurement [17]. For that reason, in this study all root canals were measured by the same file which was size 15, and one possible explanation that the two endodontists made more accurate measurements than the radiologist is their caution with regard the apical area and awareness of the repercussion of inaccurate measurement [18, 19].

Jafarzadeh *et al. *[20]*, *evaluated the conventional radiography and an electronic apex locator in determining the working length in c-shaped canals. They concluded that the apex locator was more accurate in determination of the working length of C-shaped canals compared with the conventional radiography. 

Khorasani and Ebrahimnejad [21] compared the accuracy of conventional and digital radiography in root canal working length determination in an *in vitro* study. They concluded that there was no difference between the measurement accuracy of CCD, PSP and conventional imaging techniques in root canal working length determination [21].

de Morais *etal. *[22] performed a clinical study to determine working length using cone-beam computed tomography, periapical radiography and electronic apex locator in teeth with apical periodontitis. They concluded that working length determination using CBCT images was precise when compared to radiographic method and electronic apex locator [22].

Some researchers [23] demonstrated that there were no significant differences in measurement errors among intra-oral radiographic F,E and D speed films. Even though comparing the various speed film was not our main goal, there were no significant difference between conventional film (E speed) and self-developing film (D speed) in determining the root canal working length. Apparently, the root canal morphology had more importance.

## Conclusion

According to the results, there were no significant differences between measurements of different radiographic methods. Considering each of these radiographic methods has their own particular advantages, depending on the situation whichever that is more applicable should be used.
